# Isolation, Characterization and Lipid-Binding Properties of the Recalcitrant FtsA Division Protein from *Escherichia coli*

**DOI:** 10.1371/journal.pone.0039829

**Published:** 2012-06-27

**Authors:** Ariadna Martos, Begoña Monterroso, Silvia Zorrilla, Belén Reija, Carlos Alfonso, Jesús Mingorance, Germán Rivas, Mercedes Jiménez

**Affiliations:** 1 Centro de Investigaciones Biológicas (CIB-CSIC), Madrid, Spain; 2 Instituto de Química-Física “Rocasolano” (IQFR-CSIC), Madrid, Spain; 3 Servicio de Microbiología, Hospital Universitario La Paz, IdiPAZ, Madrid, Spain; Griffith University, Australia

## Abstract

We have obtained milligram amounts of highly pure *Escherichia coli* division protein FtsA from inclusion bodies with an optimized purification method that, by overcoming the reluctance of FtsA to be purified, surmounts a bottleneck for the analysis of the molecular basis of FtsA function. Purified FtsA is folded, mostly monomeric and interacts with lipids. The apparent affinity of FtsA binding to the inner membrane is ten-fold higher than to phospholipids, suggesting that inner membrane proteins could modulate FtsA-membrane interactions. Binding of FtsA to lipids and membranes is insensitive to ionic strength, indicating that a net contribution of hydrophobic interactions is involved in the association of FtsA to lipid/membrane structures.

## Introduction

FtsA, a division protein widely conserved in bacteria, plays an essential role in cell division. In *Escherichia coli*, together with ZipA, FtsA has been proposed to be responsible for Z-ring attachment to the membrane [1], [2], [3]. A structural model of *E. coli* FtsA, built from the structure of *Thermotoga maritima* FtsA [4], shows a highly conserved C-terminal amphipathic helix potentially able to interact with the membrane, joined to the core of the protein by a flexible linker [5].

FtsA belongs to the actin/Hsp70/sugar kinase superfamily [6], [7]. Previous works showed that *E. coli* FtsA is capable to bind ATP with very low affinity, but no physiological role for this nucleotide-interaction ability has been reported so far [7], [8] and only a marginal ATPase activity has been detected [8]. Interestingly, significant ATPase activity has only been described for FtsA from *B. subtilis* [9]. No ATPase activity has been reported for FtsA from other bacteria, including that from *S. pneumoniae* [10].

Biochemical and biophysical characterization of *E. coli* FtsA has been hindered because of the protein recalcitrance to be isolated in a fully functional state. One of the most important obstacles in FtsA purification has been the lack of stability together with the tendency to aggregate. As a consequence, most *in vitro* studies so far reported on *E. coli* FtsA have been conducted with partially purified protein, cell extracts or the hypermorph mutant FtsA* [8], [11], [12].

Here we report a novel purification method improving the yield and stability of *E. coli* FtsA. The procedure, optimized from a previous protocol [13], involved the controlled refolding of the protein obtained from inclusion bodies following overproduction. This purified protein was successfully incorporated, together with FtsZ (the main protein of the Z-ring; [14]), inside giant vesicles obtained from bacterial inner membranes allowing to show that during polymerization in the presence of GTP the interaction between FtsZ monomers is stronger than the binding strength of FtsA to the membrane [15]. In this study the secondary structure, stability and association state of FtsA purified following this new protocol have been characterized using several biophysical techniques and its binding to lipids has been quantified using lipid coated micro-beads as bio-mimetic membrane models.

## Materials and Methods

### Protein Over-production and Purification

His6-FtsA was expressed as described in [8]. Pelleted cells were suspended (20 mL/L culture) in 50 mM Tris-HCl, 1 mM TCEP, 1 mM PMSF, pH 7.5 supplemented with 10 g/mL DNase, lysed by sonication and centrifuged (15 min at 10,000×*g*, 4°C). The obtained pellet was washed twice in 20 mM Tris-HCl, 10 mM EDTA, pH 7.5, 1% (v/v) Triton X-100. After removal of residual Triton X-100, inclusion bodies were solubilized in 20 mM Tris-HCl, 5 M guanidine-HCl, 0.5 M NaCl, pH 7.5 (5 mL/L culture), loaded onto a HisTrap FF column (GE Healthcare) and washed in 20 mM imidazol. An imidazole elution gradient (20–500 mM) was then performed, the eluted fractions pooled in 5 mg/mL aliquots and stored (−80°C). Refolding of FtsA was carried out by extensive dialysis against 50 mM Tris-HCl, 0.5 M NaCl, 5 mM MgCl_2_, 0.2 mM TCEP, 0.1 mM ADP, pH 8 at 4°C. Buffer was exchanged for 50 mM Tris-HCl, 0.5 M KCl, 5 mM MgCl_2_, pH 7.5 (working-buffer) with 0.2 mM TCEP and 0.1 mM ADP. Residual aggregates were removed by centrifugation (120,000×*g*, 30 min). FtsA concentration was determined by Bradford assay and by UV-absorbance (ε_280 nm_ = 33527 M^−1^ cm^−1^, estimated from quantitative amino acid analysis) with the same result.

FtsA was labeled with Alexa-488 (Invitrogen) carboxylic acid succinimidyl ester as described [15], at 0.5–0.9 labeling ratio.

### Lipid Preparations

Polar extract phospholipids from *E. coli* (Avanti Polar Lipids, Alabama, USA) in chloroform:methanol 1∶1 (v/v) were dried under nitrogen flow and kept under vacuum for at least two hours. Multilamellar vesicles (MLVs) were obtained by hydration of the dried film in 50 mM Tris-HCl, 0.5 M KCl, pH 7.5. Large unilamellar vesicles (LUVs) were formed by extrusion from these MLVs as previously described [16]. Bacterial inner membrane vesicles (IMVs) were isolated from wild-type *E. coli* cultures (JM600 strain) grown to exponential phase [17] as described [15], [18].

### Fluorescence Measurements

The emission spectra of 1 µM unfolded and folded FtsA were acquired on a PC1 spectrofluorometer (ISS), using 3×3-mm pathlength quartz cuvettes (Starna Hinault), at λ_exc_ = 295 nm, 20°C in working-buffer with and without 5 M guanidine-HCl, respectively.

### Sedimentation Velocity

Experiments were conducted at 48,000 rpm and 10°C in an XL-I analytical ultracentrifuge (Beckman-Coulter Inc.) equipped with a UV-VIS detection system, an An-50 Ti rotor and 12 mm double-sector centerpieces. Sedimentation coefficient distributions of 2.1–10.5 µM FtsA in working-buffer were calculated using SEDFIT [19]. *s*-values were corrected to standard conditions (water, 20°C) using SEDNTERP [20]. For monomeric FtsA, theoretical sedimentation coefficients were calculated with HYDROPRO [21] using a homology model (residues 7-385) based on the structure of *T. maritima* FtsA [4] [22].

### Circular Dichroism

Far-UV CD spectra were recorded in a JASCO J-810 spectropolarimeter equipped with a Peltier type cell holder (Jasco Corp.) in 0.1 cm pathlength cells at 2.5 µM FtsA in working-buffer with and without 5 M guanidine-HCl. Thermal denaturation (5–75°C, heating rate 20°C/h) was followed at 222 nm. Ellipticities corrected for buffer contribution were converted to mean residue ellipticities using a mean molecular mass per residue of 108.

### ATPase Activity and Nucleotide Binding

ATPase activity was monitored as described in [23]–[24] by measuring released inorganic phosphate using the malachite green-molybdate reagent at two minutes intervals. The color developed in the sample (5 µM FtsA +10 µM ATP) and the control (5 µM FtsA) was measured for a total time of 30 minutes.

Nucleotide binding by FtsA was tested using two different assays, namely Mant-nucleotide binding and affinity chromatography. Due to the environmental sensitivity of Mant fluorescence, nucleotide analogs incorporating this fluorophore are very useful to prove nucleotide binding to proteins [25]. Samples containing 3–7 µM FtsA were added to Mant-ATP or Mant-ADP in concentrations ranging 50–100 µM. The fluorescence emission of Mant-ATP or Mant-ADP upon excitation at 356 nm was measured after 30 minutes (or 1 h) incubation, either at 4°C or at room temperature. The retention of *E. coli* FtsA in EDA-ADP (Jena Biosciences) or ATP-agarose columns (ATP affinity test kit, Jena Biosciences) was tested following manufacturer’s specifications.

### Lipid Interaction Assays

Turbidity assays were conducted using an Ultrospec3000-*Pro* spectrophotometer (GE Healthcare) using 1 cm quartz cuvettes (Hellma) at 350 nm. Phospholipid vesicles (0.2 g/L) in working-buffer were incubated at room temperature with or without 2 µM FtsA.

Lipid coated silica beads (4.63 µm diameter, Bangs Laboratories) were prepared following a protocol to be described elsewhere (Monterroso *et al*., in preparation). Briefly, beads were incubated with an excess of coating material (MLVs or IMVs, 1 h, room temperature), successively pelleted and washed. Concentration of accessible lipid on coated beads was calculated, assuming a single bilayer, as half of the lipid quantified as described [26]. A 20% lipid in the outer leaflet was assumed for IMVs [27]. Binding isotherms were built from fluorescence emission (λ_exc_ = 495 nm; λ_em_ = 520 nm) of FtsA-Alexa488 remaining in the supernatant after centrifugation of 200 nM FtsA-Alexa488 incubated with increasing concentrations of beads. The linearity of FtsA-Alexa488 emission with FtsA concentration was verified. Labeled protein was used because of the negligible background contribution in the samples.

Protein binding to lipid bilayers can be described defining an apparent association constant, *K* (the reciprocal of the lipid concentration at which half of the protein is bound) that does not require any assumptions about the adsorption mechanism [28]:
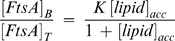
(1)where [*FtsA*]*_B_*, [*FtsA*]*_T_*, [*lipid*]*_acc_* are the concentrations of bound and total FtsA and accessible lipids, respectively. Equation (1) was fitted to the recovered isotherms using user-written functions in MATLAB (Ver. 7.10, MathWorks).

## Results

### Purification of *E. coli* FtsA from Inclusion Bodies

**Figure 1:**
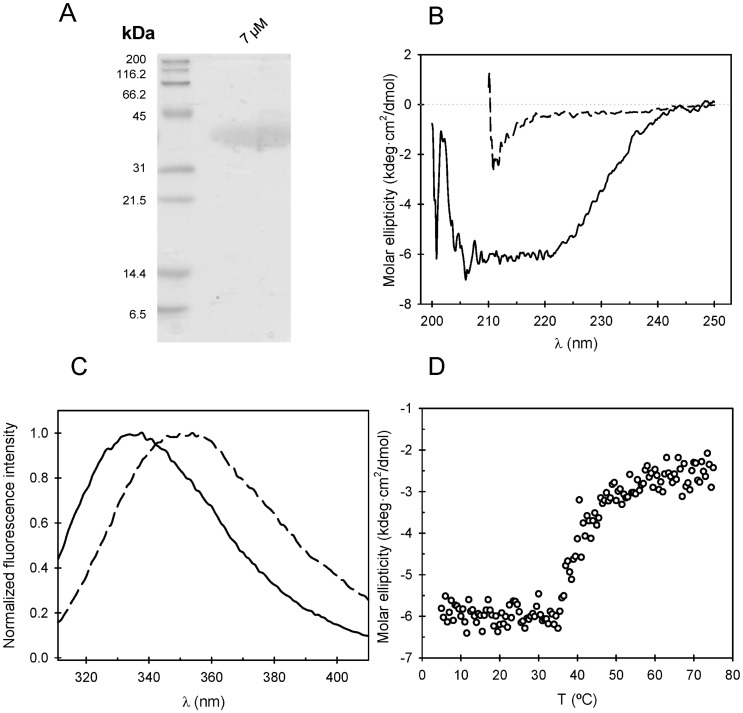
Molecular characterization of purified *E. coli* FtsA protein. (A) SDS-PAGE analysis. Loaded FtsA concentration was 7 µM. Molecular weight markers are in the left lane. (B) Far-UV circular dichroism spectra (CD) of denatured (dashed) and refolded FtsA (solid). (C) Corrected fluorescence emission spectra of denatured (dashed) and refolded (solid) FtsA. λ_exc_ = 295 nm, 20°C. (D) Thermal unfolding of FtsA as monitored by CD.

The yield obtained with the new protocol of purification was nearly 15 mg FtsA over 95% pure per L of culture, as judged by SDS-PAGE (**Figure 1A**), displaying an electrophoretic migration compatible with the size expected for monomeric FtsA (47.6 kDa). Our new procedure presents enhanced yield and higher level of purity and reproducibility of FtsA solutions with respect to previously published protocols.

Upon refolding, far-UV CD spectrum of FtsA shows a gain of secondary structure typical of α/β proteins (**Figure 1B**) that is fully compatible with that of FtsA purified form the soluble fraction by Yim and coworkers [8]. This is accompanied by a blue shift in the fluorescence emission spectrum **(** **Figure 1C** **)**, consistent with an increase in the hydrophobicity of the environment of the tryptophan residues harbored by the protein, pointing at a gain of folded tertiary structure. Moreover, the CD thermal unfolding profile of FtsA (**Figure 1D**) shows a transition between the native and denatured states at around 41°C, indicating the protein contains tertiary contacts characteristic of a folded protein. Neither significant ATP/ADP binding nor ATPase activity were detected (see Materials and Methods).

### FtsA is Mostly Monomeric in Solution

**Figure 2:**
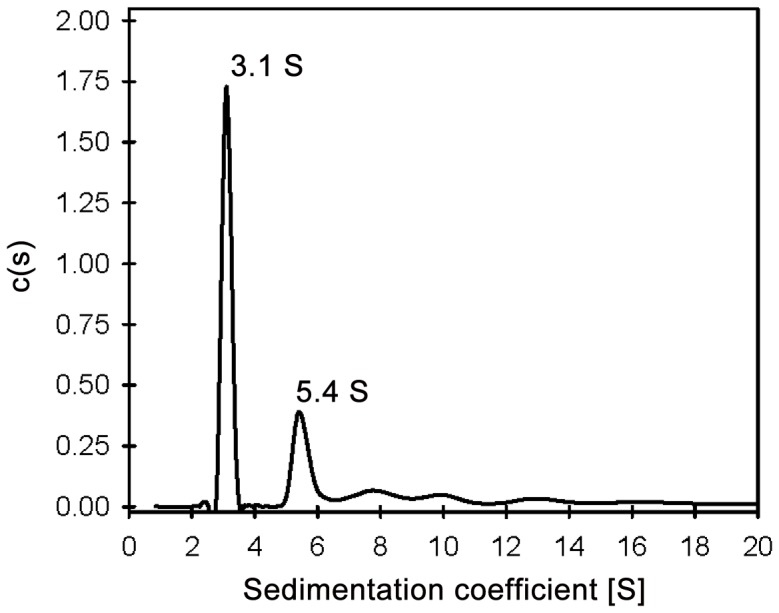
Sedimentation coefficient *c(s)* distribution of *E. coli* FtsA (10 µM) in working buffer with 0.1 mM ADP and 0.2 mM TCEP, generated from the sedimentation velocity interference data.

Sedimentation velocity allowed determining that the final purified FtsA protein exists mainly as a monomer in solution (**Figure 2**). Sedimentation coefficient *c(s)* distribution analysis of FtsA (10 µM) showed that most of the protein (>85% of the loading concentration) sedimented as a 3.1 S species, compatible with the theoretical *s*-value (2.8 S) for monomeric FtsA (see Materials and Methods). A 5.4 S species (ca. 5%), compatible with FtsA dimers or trimers, and minimal amounts of species with higher *s*-values, compatible with protein oligomers, were also observed. A concentration-dependent study to characterize the nature of these oligomers was precluded as the protein did not remain soluble at concentrations >15 µM. The sedimentation behavior of FtsA was not affected by the presence of 1 mM ADP (or ATP). Fluorescence cross-correlation spectroscopy measurements were also compatible with monomers as the main species populating FtsA solutions (**Figure S1**).

### FtsA Interacts with Inner Membranes with Higher Apparent Affinity than with *E. coli* Lipids

Interaction of FtsA with lipid structures was first explored by conducting a rapid assay in which the turbidity of a solution containing large unilamellar lipid vesicles (LUVs) was monitored before and after adding FtsA. A fast absorbance increase indicative of FtsA interaction with lipids, leading to vesicle aggregation, was observed when FtsA was added into a solution containing LUVs made from *E. coli* lipids. In contrast, no such turbidity rise was detected in LUVs made from inner membrane vesicles (**Figure 3A**). The lack of significant turbidity enhancement in the solutions of inner membrane vesicles upon addition of FtsA indicated that no detectable vesicle aggregation occurred in the presence of the protein, but it did not rule out interaction between FtsA and the inner membrane (see below). Induction of liposome aggregation, on the other hand, has been previously reported for other lipid binding proteins [29]–[30] and it is often linked to the presence of more than one lipid binding domain either harbored by a single protein molecule or by an oligomeric species.

**Figure:**
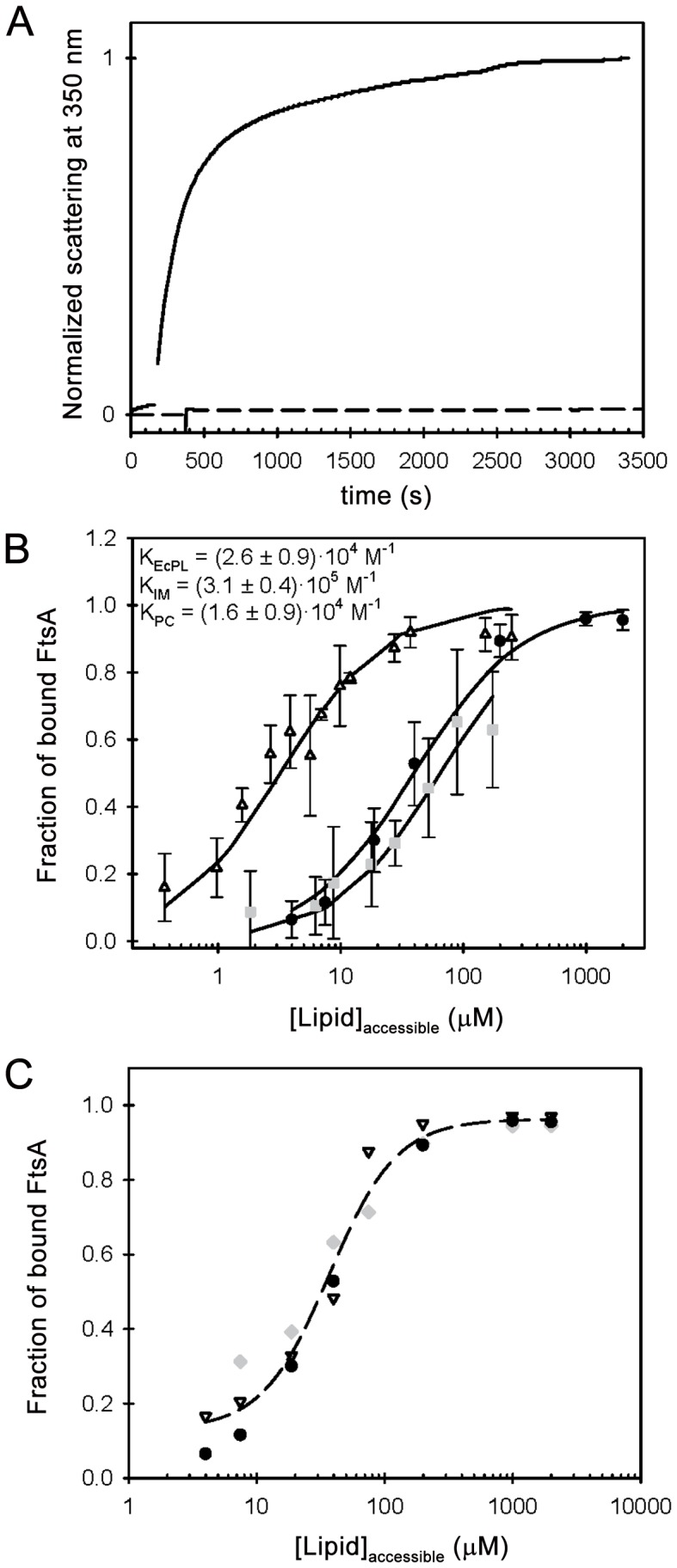
Interaction of *E. coli* FtsA with lipid/membrane structures. (A) FtsA binding to large unilamellar liposomes made of *E. coli* phospholipids (solid) or inner membrane vesicles (dashed) as monitored by turbidity. (B) FtsA binding to micro-beads coated with *E. coli* lipids (circles), inner membrane (triangles) and phosphatidylcholine (squares). Data points correspond to the average of at least three individual measurements ± SD. Solid lines represent the best fit of equation (1) to the data with the best-fit parameters shown in the figure. (C) Effect of ionic strength on FtsA binding to EcPL-beads. 100 (circles), 300 (triangles) and 500 mM KCl (diamonds). Errors bars are omitted for the sake of clarity. Dashed line is just intended to guide the eye.

To quantify the interaction of FtsA with lipids, we performed co-sedimentation assays with silica micro-beads coated with the *E. coli* lipids (EcPL-beads), phosphatidylcholine (PC-beads) or *E. coli* inner membrane (IM-beads). FtsA was found to interact with the three kinds of lipid-coated beads, and the level of interaction was not sensitive to the presence of 1 mM concentration of either ATP or ADP. The apparent affinity of the interaction with the beads coated with inner membrane was at least ten-fold higher than with those coated with *E. coli* lipids (**Figure 3B**). Interestingly, the apparent affinity of FtsA for micro-beads coated with phosphatidylcholine was only slightly lower than when the *E. coli* lipids mixture was the coating material of the beads, suggesting a contribution of hydrophobic interactions on FtsA binding to lipids. This conclusion was further supported by a lack of influence of ionic strength on the interaction between FtsA and beads coated with *E. coli* lipids (100–500 mM KCl, **Figure 3C**). It is noteworthy that, at high lipid concentrations, no residual protein was detected in the supernatants, meaning that purified FtsA fully retained lipid-binding activity.

## Discussion

Here we present a new method for the purification of *E. coli* FtsA. Our results confirm that purified FtsA is folded, as it presents the spectroscopic features of the previously reported FtsA purified in its folded native state [8], its thermal unfolding profile is that expected for a folded protein, and no significant amount of FtsA incompetent for lipid binding was found in the solutions. Purification of FtsA has proved to be very complicated due to the amphitropic nature of the protein. The difficulty is further enhanced due to the low amounts of FtsA protein in *E. coli* cells, compared with that of other division proteins such as FtsZ [31], hampering its isolation from the soluble fraction. The only protocol available so far rendered *E. coli* FtsA purified from the soluble fraction of the homogenate by metal affinity chromatography on a cobalt column [8]. The protein purified following this protocol was highly unstable (J. Mingorance, personal communication), which hindered a thorough biophysical characterization of the material. In our hands, compared with the previous purification protocol from the soluble fraction [8], FtsA solutions prepared according to the new procedure displayed less tendency towards aggregation and the presence of protein and lipid contaminants became tremendously reduced to almost undetectable levels. On the other hand, the yield and concentration of FtsA in the solutions achievable with this new method considerably exceeded those previously attained, thus allowing for a better biochemical and biophysical characterization of the protein.

To set up our new procedure to purify FtsA from *E. coli* we used a previously published protocol for the purification of *P. aeruginosa* FtsA [13] as a starting point. Several modifications to this protocol were critical: i) In the original protocol, inclusion bodies of *P.* *aeruginosa* FtsA were solubilized using CAPS (*N*-cyclohexyl-3-aminopropanesulfonic acid) buffer (a zwitterionic buffer used for pH 7.9–11.1) with N-lauroylsarcosine. In order to remove the detergent, an additional step using an anion exchange resin was required. The strong interactions of *E. coli* FtsA with column matrixes prompted us to avoid detergent addition, which would have been hard to remove, as a means to solubilize inclusion bodies. We achieved solubilization of inclusion bodies with 5 M guanidine-HCl, a denaturant agent selected for being more stable than urea solutions. ii) Besides, we kept the buffer pH between 7.5–8 using 20 mM Tris-HCl, 0.5 M NaCl and, due to instability of DTT included in the original protocol, we chose TCEP instead. iii) Refolding of FtsA was carried out by a multistep dialysis procedure of solubilized inclusion bodies against 100 volumes of refolding buffer. The buffer composition rendering optimal refolding was empirically determined by a step by step testing of different components and contents. The main difference regarding the original protocol was the presence of high concentrations of NaCl, 5 mM MgCl_2_ and ADP, all of them absolutely required for proper refolding. FtsA formed visible aggregates in the absence of NaCl or ADP and with lower or higher amounts of Mg. The time required for each dialysis step within a refolding procedure in the original protocol was also optimized, decreasing from 5–12 hours to 3 hours.

Despite belonging to the actin/Hsp70/sugar kinase superfamily [6]–[7] and ADP being necessary to achieve successful refolding, no nucleotide binding or ATPase activity were detected in the purified FtsA. We cannot rule out low affinity interactions or the presence of ADP from the refolding step already incorporated in FtsA. However, the tendency of the protein to aggregate at concentrations higher than 20 µM precludes the detection of bound nucleotide in the presence of high concentrations of free nucleotide (in the mM range) conditions required to measure binding properties in weak ligand-receptor systems. Furthermore, lipid/membrane binding was insensitive to millimolar concentrations of ATP or ADP stating that nucleotide binding does not seem to mediate recognition. Several facts agree with our results since only *B. subtilis* [9] and *Pseudomonas aeruginosa* FtsA [13] have been reported to display measurable ATPase or phosphatase activity, respectively; a very marginal activity has been described for *E. coli* FtsA* hypermorph [32]; no ATPase activity has been detected for *Thermotoga maritima* FtsA [33]; and very weak ATP binding has been reported for *E. coli* FtsA, obtained from the soluble fraction [8] and by photoaffinity labeling in cellular extracts [7].

According to our results, the protein interacts differently with *E.* *coli* lipids and with the inner membrane, being the apparent affinity higher for the membrane (micro-bead assays). Besides, FtsA interaction with PC, a zwitterionic phospholipid, together with the nonexistent effect of ionic strength on its interaction with *E. coli* lipids strongly suggests that FtsA binding to lipids is not preferentially electrostatic. The higher affinity of FtsA for the inner membrane might arise from specific contributions of one or more of the proteins within this structure. Indeed, the interaction of FtsA with some of the late divisome proteins is well known, being essential for their recruitment [31]. Another possibility is that the affinity of FtsA for the lipids themselves is enhanced in the configuration they adopt in the membrane environment.

FtsA interaction with lipids has been predicted to occur through its C-terminal helix, the only lipid-binding domain identified so far [34], suggesting that aggregation may be coupled to FtsA self-association. Indeed, although the major species found in the sedimentation velocity experiments is compatible with FtsA monomer, small amounts of oligomers were detected, in agreement with previous observations that pointed out the ability of FtsA to self-interact through its C-terminus to form dimers and perhaps higher order oligomers [22] [35]–[36]. Moreover, it has been proposed that self-association or assembly of FtsA may be promoted by membrane binding [34].

In the Supporting Information it is shown that the FtsA protein purified by the procedure described in this work does not polymerize in the absence of lipids, whereas it does form polymer structures on lipid/membrane-coated micro-beads (scanning electron microscopy analysis, Figure **S2**) and on lipid monolayers (electron microscopy analysis, **Figure S3**). These results are consistent with the finding that FtsA from *Thermotoga maritima* forms polymers *in vitro* on lipid monolayers, but not in the absence of lipids [33].

In summary, the purification protocol reported here has allowed to obtain milligram amounts of well-behaved FtsA division protein from *Escherichia coli* and to measure the lipid-binding properties of FtsA relevant for its functional activity.

## Supporting Information

Figure S1 **Autocorrelation and cross-correlation curves obtained from two-color fluorescence.** cross-correlation spectroscopy (TCFCCS) measurements of a mixture of FtsA labelled with Atto 647N and with Alexa 488.
(EPS)

Figure S2 **Scanning electron microscopy analysis of FtsA polymers formed in the presence of lipid/membrane-coated beads.**
(EPS)

Figure S3 **Electron microscopy analysis of** ***E. coli*** **FtsA polymers formed on lipid monolayers.**
(EPS)
